# AAV Delivery of shRNA Against TRPC6 in Mouse Hippocampus Impairs Cognitive Function

**DOI:** 10.3389/fcell.2021.688655

**Published:** 2021-07-13

**Authors:** Ruxin Xie, Zhongke Wang, Tianyao Liu, Rui Xiao, Keyi Lv, Chuan Wu, Yi Luo, Yun Cai, Xiaotang Fan

**Affiliations:** Department of Military Cognitive Psychology, School of Psychology, Third Military Medical University (Army Medical University), Chongqing, China

**Keywords:** TRPC6, hippocampus, cognitive process, synapse, shRNA

## Abstract

Transient Receptor Potential Canonical 6 (TRPC6) has been suggested to be involved in synapse function and contribute to hippocampal-dependent cognitive processes. Gene silencing of TRPC6 was performed by injecting adeno-associated virus (AAV) expressing TRPC6-specific shRNA (shRNA-TRPC6) into the hippocampal dentate gyrus (DG). Spatial learning, working memory and social recognition memory were impaired in the shRNA-TRPC6 treated mice compared to control mice after 4 weeks. In addition, gene ontology (GO) analysis of RNA-sequencing revealed that viral intervention of TRPC6 expression in DG resulted in the enrichment of the process of synaptic transmission and cellular compartment of synaptic structure. KEGG analysis showed PI3K-Akt signaling pathway were significantly down-regulated. Furthermore, the shRNA-TRPC6 treatment reduced dendritic spines of DG granule neurons, in terms of spine loss, the thin and mushroom types predominated. Accompanying the spine loss, the levels of PSD95, pAkt and CREB in the hippocampus were decreased in the shRNA-TRPC6 treated animals. Taken together, our results suggest that knocking down TRPC6 in the DG have a disadvantageous effect on cognitive processes.

## Introduction

The transient receptor potential channels (TRPCs) comprised of seven different channels (TRPC1-TRPC7), are calcium-permeable, non-selective cation channels containing six transmembrane structural domains (Dietrich et al., 2005; Nakata and Nakamura, 2007; Venkatachalam and Montell, 2007). Recent studies have revealed that TRPCs are broadly expressed in the central nervous system (CNS), and critical for fundamental cellular events ranging from neuronal growth, synaptic development to neuronal differentiation (Gomez et al., 2001; Jia et al., 2007; Tai et al., 2009). Among TRPCs, the TRPC6 channel is emerging as a prominent target for the control of synapse formation involved in the cognitive function (Zhou et al., 2008). Though many aspects of the physiology and regulation of TRPC6 are still elusive, dysfunction of the TRPC6 channel may trigger a wide range of CNS-related diseases such as epilepsy, autism spectrum disorder (ASD) and Alzheimer’s disease, characterized by cognitive dysfunction (Griesi-Oliveira et al., 2015; Kim and Kang, 2015; Thapak et al., 2020).

The hippocampus is involved in learning, memory formation, spatial coding, and mood regulation (Douglas, 1967; Eichenbaum, 2017). The dentate gyrus (DG) is one of two known brain regions producing new neurons throughout life. Synapse and dendrite abnormalities occurred in DG granule cells (DGCs) are associated with altered learning and memory in both humans and mice. Particularly, it has been indicated that the DG also regulates pattern separation, which differentiates related memories by transforming similar input firing patterns into distinct output firing patterns via granule cells (Hainmueller and Bartos, 2020). Previous studies have shown that TRPC6 is expressed in pyramidal neurons, DGCs, interneurons and some glial cells in the hippocampus (Chung et al., 2006; Tai et al., 2008). TRPC6 knockdown leads to the massive DGC degeneration following status epilepticus. TRPC6 protein is enriched in synaptosomal and postsynaptic fractions, mainly concentrated in the postsynaptic fraction. It has been confirmed that TRPC6 is critical for excitatory synapse formation, which is further supported by the finding that TRPC6 overexpression could increase dendritic spine density (Zhou et al., 2008).

The purpose of this present study was carried out to test the hypothesis that down-regulation of TRPC6 in the hippocampus DG leads to cognitive dysfunction. To do so, we made use of adeno-associated viral (AAV) expressing short hairpin RNA (shRNA) targeting TRPC6 into the hippocampal DG of male mice. We evaluated the TRPC6 silencing effect using a battery of cognitive behavioral examinations linked to hippocampus. High-throughput RNA-sequence (RNA-seq) was conducted to identify differential expression genes (DEGs) which can elucidate the potential mechanism of regulating the hippocampus related function. We uncovered a major role for TRPC6 in the DG in synapse function that are involved in cognitive function.

## Materials and Methods

### Animals

Adult Male C57BL/6J mice (6–8-week-old, 22–28g) were provided by Lepitec Biotechnology Ltd. (Chongqing, China). The animals were divided into 4–6 mice per cage kept in a controlled environment (22 ± 2°C, 45 ± 10% humidity, 12 h light/dark cycle) and have free access to food and water. All experimental procedures are carried out in accordance with the standards approved by the Laboratory Animal Ethics Committee of the Army Medical University, while ensuring that animal suffering is alleviated. The document approval number is AMUWEC20211149.

### Stereotactic Injection

The shRNA was used to silence the expression of TRPC6. Custom-made AAV vectors carrying shRNA targeting mouse TRPC6 (shRNA-TRPC6) and control AAV-CMV-EGFP (shRNA-control) were purchased from Taitool Biotechnology Co., Ltd. (Shanghai, China). Animals were randomly divided into shRNA-control and shRNA-TRPC6 groups and code labeled by an independent researcher. After a 1-week acclimatization period, the mice were fixed in the stereotaxic apparatus (RWD instruments) and anesthetized with isoflurane (3.0% for induction and 1.5% for maintenance). Using an automatic microinjection system (World Precision Instruments), shRNA-control and shRNA-TRPC6 were bilaterally injected into the hippocampus DG region according to the following coordinates: AP = −1.9; ML = ± 1.1; DV = − 2.0. Injections were performed at a speed of 0.1 μL/min with Hamilton needle (1 μL, 7.5 × 10^12^ viral particles per mL), the needle was left in place for 10 min before slowly retracted (Liu et al., 2017). Behavioral tests were conducted 4 weeks after virus injection.

### Behavioral Assays

#### Nest Building Task

Nest building task was performed as described previously and the quality of the nests was scored with minor modifications (Deacon, 2006). shRNA-TRPC6 injected mice and control mice were placed in a clean standard cage individually, and a square of compressed cotton (Ancare, United States) nesting material of nearly 2.5 g/5 cm^2^ was introduced in the middle of the cage and monitored for 12 h. After 12 h, the quality of nests built was recorded on a five-point scale to evaluate nesting behavior.

#### Y Maze Test

To test the short-term spatial working memory capacity (by spontaneous alternation) of mice, spontaneous alternating behavior was tested using a customized Y-maze. Three compartments (30 cm long, 5 cm wide and 15 cm high) at the same angle (120°) form the structure of the Y-maze. The mice were placed individually in the center of the triangle of the maze and explored freely for 8 min. The automatic tracking system (Ethovision 11.5 software, Noldus Information Technology) monitored and counted the total number of times (N) each mouse enters the arm and the number of correct alternations (N1). A correct alternation is defined as a mouse entering the three different arms in sequence, in detail, an entry that is different from the first two adjacent entries. Alternation rate (%) = N1/(N − 2) × 100%.

#### Novel Object Recognition (NOR)

To assess short-term memory, the NOR test was carried out as previously described, with slight modifications (Ennaceur and Delacour, 1988). As the mouse is naturally attracted to novelty, it will spend most of its time on the new object after recognizing the familiar one. The whole experiment took a total of 30 min, which was divided into three 10-min phases. In the final phase, the time spent exploring each object was recorded (N, time to explore new objects; F, time to explore familiar objects) and the recognition rate was calculated (N/(N + F) × 100%). The trajectory of each mice’s movement during the experiment was analyzed using ethovision 11.5 (Noldus) software.

#### Open-Field Test (OFT)

General exploratory locomotion in the open field apparatus was evaluated according to our previous described (Zhang et al., 2019). During the test, a mouse was placed in the center of open field box and allowed to explore for 30 min. The total distance moved (as a measure of locomotor activity) and the duration in the central area (as a measure of anxiety-like behavior) were analyzed with Ethovision 11.5 (Noldus) software.

#### Three-Chamber Sociability Test

The three-chamber sociability test was performed as described previously (Cai et al., 2018). The social interaction test consists of three 10-min phases. In the first phase, mice were allowed to freely acclimatize to the site for 10 min. In the second stage, a stranger mouse (S1) and a novel object (Dietrich et al., 2005) were placed in the left and right chamber. Subject mice were then allowed to freely explore the apparatus for 10 min. In the third stage, the novel object was replaced with a new stranger mouse (S2). The test mice explored for 10 min before ending the experiment. Noldus Observer software (Ethovision 11.5) was used to assess the amount of time spent in each of the three chambers. Moreover, social preference levels which represented the numerical difference between the time in the chambers and sniffing (S1 vs. object or S2 vs. S1) divided by total time, were also determined.

#### Morris Water Maze Test

The Morris water maze (MWM) test was conducted as described previously (Cai et al., 2018; Li et al., 2018). Briefly, the MWM was a circular pool of 120 cm in diameter and 50 cm height, filled with water mixed with non-toxic white paint. The pool was virtually divided into 4 equal quadrants. A white circular platform of 12 cm in diameter was submerged 1 cm under the opaque water surface, invisible for the mice at the water level, was located in a fixed quadrant center. For 5 consecutive days, mice performed 4 trials per day (60 s/trial) to find a hidden escape platform. The start location was randomly assigned between 4 quadrants of the pool. If the mice did not reach the hidden platform within the allotted time, the trial was terminated and the mice were carefully guided to the platform. After the mice climbed onto the platform, they were left on it for 30 s and then removed from the pool. The mice were then dried and returned to their home cages until the next test. On day 7, the evacuation platform was removed and probing was performed. On day 8th, the reverse MWM test was performed.

In the reversed MWM test, the reversed test task was performed. An average of the escape delays from the 4 trials on each day was taken. On day 11, the platform was removed and a 60 s trial was conducted. The escape latencies, the time in target quadrant and distance were recorded and analyzed with Ethovision 11.5 software.

### Immunofluorescence

**FIGURE 1:**
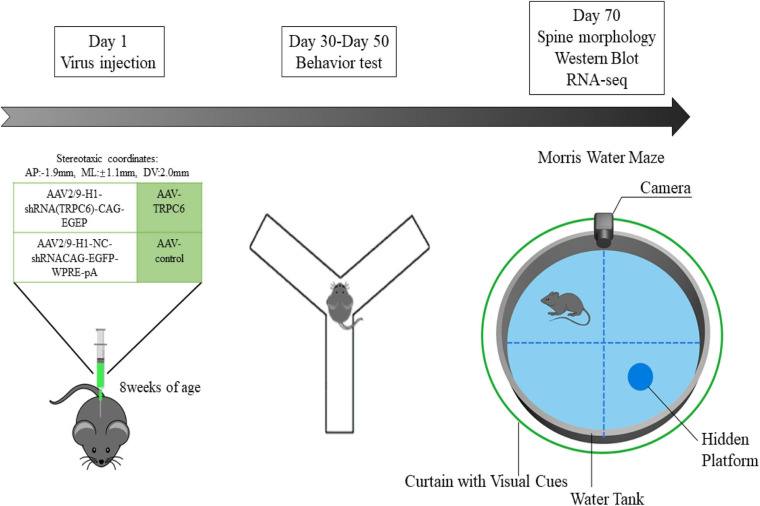
Scheme of the expe rimental design. In the first experimental phase, we administered different viral injections to two groups of mice to explore the role of TRPC6 in the DG region of the hippocampus. In the second experimental phase, also 28 days after virus injection, the animals were used for behavioral studies on Nest building, Y-maze, NOR, Open field, Three-chamber sociability and Morris water maze. Later, in the third experimental phase, we performed Western blotting, qRT-PCR, RNA-seq, immunofluorescence and spine morphology studies.

After the behavioral experiments, some of the mice from both groups were sacrificed and the pathological changes in mice were examined by immunofluorescence technique (Figure 1). According to previously described (Liu et al., 2016), the mice were anesthetized and perfused with saline followed by 4% paraformaldehyde (PFA). The brain tissue was placed in 4% PFA solution for post-fixed for 24 h and stored in 30% sucrose solution for 24 h. The brains were cut coronally into 30 μm-thick sections by using a cryostat. The sections were permeabilized in PBS containing 0.5% Triton X-100, 3% H_2_O_2_ for 30 min, then incubated in 3% BSA for 30 min. Briefly, the sections were then incubated with rabbit anti-TRPC6 antibody (1:200; Millipore Corporation, Billerica, MA, United States) for 12 h at 4°C. Followed by incubation with Cy3-conjugated secondary antibody (1:500, Jackson ImmunoResearch) for 2 h at room temperature. Images were captured with a Zeiss microscope (Carl Zeiss, Jena, Germany).

### RNA Extraction, Library Construction, and Sequencing

Fresh tissue from mice hippocampus was extracted from total RNA using Trizol kit (Invitrogen, Carlsbad, CA, United States). The quality of RNA was assessed by Agilent 2100 Bioanalyzer (Agilent Technologies, Palo Alto, CA, United States) and also checked by RNase-free agarose gel electrophoresis. After extraction of total RNA, it was enriched using Oligo (dT) beads and reverse transcribed into cDNA using random primers. After synthesis of the second strand, the cDNA was purified, end-repaired and added to Illumina sequencing adapters. The size of the ligated product was selected by agarose electrophoresis, and after PCR amplification, sequencing was performed using Illumina HiSeq2500 by Gene *Denovo* Biotechnology Co. (Guangzhou, China).

### Mapping QC and Data Analysis

For each gene, the expected number of Fragments Per Kilobase transcribed sequence base pairs (FPKM) based on gene length and read count was calculated and used as an indicator of the gene expression level. For the analysis of differentially expressed genes (DEGs), conventional statistics were performed using the DESeq R package (1.18.0) with a model based on a negative binomial distribution. *P*-values < 0.05 were considered significant. DEGs were then analyzed by Gene Ontology (GO) enrichment pathways using the GOseq R package, which corrects for gene length bias based on Wallenius hyper-distribution. Enrichment of the screened differentially expressed genes for tissue type, molecular function, cellular composition and pathway was performed using the Enrichr web server tool. The smallest *p*-value indicates the highest degree of enrichment. Volcano and heat maps were plotted using the volcanoPlot and pheatmap functions in the R language.

### Quantitative RT-PCR (qRT-PCR)

Fresh hippocampal tissue was collected after behavioral studies, total mRNA (700 ng) was extracted using Trizol (Invitrogen, United States) and reverse transcribed into cDNA according to the manufacturer’s protocol. To compare the differences in gene expression levels, the cDNAs (3 μL) were amplified in qRT-PCR using the following primers: mouse Slc24a2 (forward, 5′-GGG TTC CGC CGT GTT CAA TA-3′; reverse, 5′-AGG ACA CAT CCC GAA AGA GC-3′); mouse Acvr1c (forward, 5′-ACA CTT GTG CCA TAG CTG ACT-3′; reverse, 5′-TCT GGG GTA TGT CTA TAG TGT TCA-3′); mouse Adamts2 (forward, 5′-GGA CTG TCC CAA TTC CCT GG-3′; reverse, 5′-GTG CAC CAT GCG CTT CAT AG-3′); mouse Col6a2 (forward, 5′-TGG CCC CTA ACA GGA ACC TA-3′; reverse, 5′-GGT GTC CTG GTC AAT CTC GG-3′); mouse Aldh1a2 (forward, 5′-AAT CGC TTC TCA CAT CGG CA-3′; reverse, 5′-CTC TTG GCC CTT TCC ACA CT-3′); mouse Slc6a13 (forward, 5′-CTG TGG CAT CCC TGT CTT CTT-3′; reverse, 5′-CAG ATT CTC CTC CAG GCT GT-3′); mouse TRPC6 (forward, 5′-GAT AAG TGG GAC CCT ACC G-3′; reverse, 5′-TCA TAA AGG CTA CAA ACA CC-3′); mouse TRPC3 (forward, 5′-AGG ATC AAT GCC TAC AAG G-3′; reverse, 5′-CAA GCA GAC CCA GGA AGA T-3′). Mouse β-actin (forward, 5′-TCA TCA CTA TTG GCA ACG AGC-3′; reverse, 5′-AAC AGT CCG CCT AGA AGC AC-3′) was used as internal controls. The relative levels of mRNA were detected by using the 2^ΔΔ^Ct method.

### Western Blotting

In the meantime, while preparing immunofluorescent brain slices, some mouse brains were immediately removed and the hippocampus was carefully dissected on ice for western blot experiments. Hippocampus tissue was weighed, sectioned in RIPA buffer (Beyotime, Shanghai, China) and frozen PBS, and homogenized in PMSF supplemented with a broad-spectrum phosphatase inhibitor (Xiao et al., 2017). Samples were then centrifuged at 13,000 r/min for 15 min at 4°C. Supernatants were collected and stored at −80°C. The protein concentration of the lysate was measured using a BCA analysis kit (Beyotime Institute of Biotechnology, Shanghai, China). Briefly, samples were separated by 10% SDS-PAGE (80 V, 30 min; 120 V, 80 min) and then transferred to PVDF membranes at a constant current of 220 mA for 150 min. The membranes were closed with 3% bovine serum albumin (BSA) and then incubated with rabbit anti-PSD95 (1:1,000, Cell Signaling Technology), rabbit anti-TRPC6 (1:1,000, Millipore Corporation, Billerica, MA, United States), rabbit anti-Akt (1:500, Cell Signaling Technology), rabbit anti-p-Akt (1:500, Cell Signaling Technology), rabbit anti-CREB (1:500, Cell Signaling Technology), rabbit anti-Synapsin (1:1,000, Cell Signaling Technology) and rabbit anti-Synaptopsin (1:1,000, Cell Signaling Technology) at 4°C overnight. After washing and incubation with secondary antibodies (anti-rabbit IgG 1:3,000; anti-mouse IgG 1:5,000) for 2 h at room temperature, enzyme-linked chemiluminescence was developed. Finally, band intensity was quantified in Image Lab (Bio-Rad Laboratories, United States). The relative intensities associated with mouse anti-GAPDH (1:2,000, Cell Cwbio, China) were quantified and then normalized to control values. Each experiment was repeated 3 times. 4 animals per group were statistically analyzed.

### Golgi-Cox Staining

Brains were processed for Golgi staining with the Rapid Golgi Kit (FD Neuro-Technologies, MD, Cat#PK401) according to the manufacturer’s protocol. Transverse hippocampus sections (80 μm) were generated and mounted in DPX after drying. Images were blinded and spines were manually counted and sorted as previously described (Xiao et al., 2017).

### Statistical Analysis

All data were analyzed using SPSS 25.0 and presented as mean ± standard error of the mean (SEM). The three-chamber sociability test used the statistical method of paired *t*-test. Data from MWM tests were analyzed using repeated measures two-way analysis of variance (ANOVA). Independent *t*-tests were used for the rest of the data. A *p* < 0.05 was considered statistically significant.

## Results

### Injection With shRNA TRPC6 Resulted in Decreased TRPC6 Level in the DG

**FIGURE 2:**
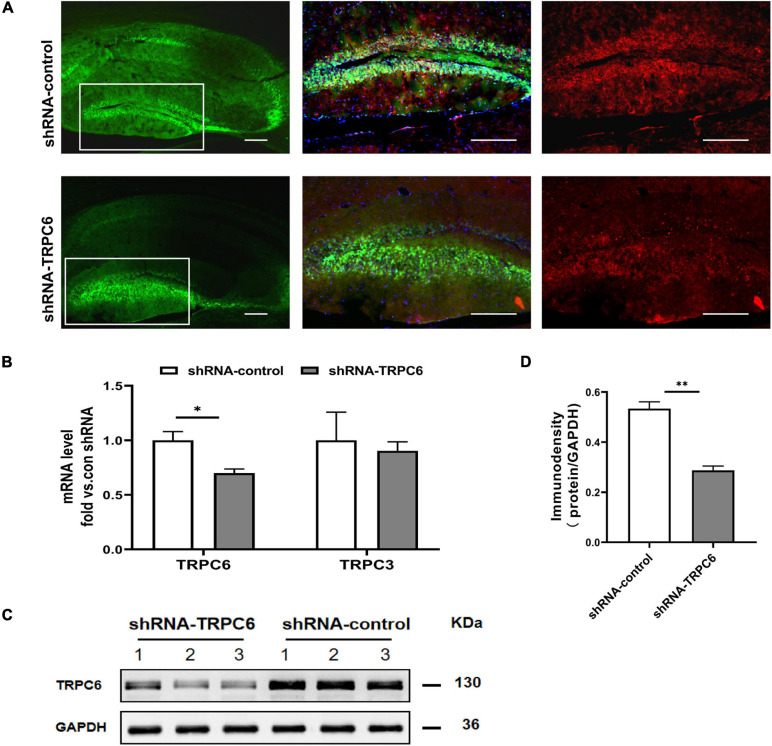
shRNA-TRPC6 injection down-regulated TRPC6 expression in the hippocampus of animals. **(A)** Double immunofluorescent staining for TRPC6 (red) and AAV expressions (green) in the hippocampus of control shRNA- and TRPC6 shRNA- injected mice. **(B)** TRPC6/TRPC3 mRNA expression in shRNA-control and shRNA-TRPC6 treated mice by qRT-PCR. **(C)** Representative image of western blotting for TRPC6 protein in hippocampus of two groups of mice. **(D)** Densitometric quantification of TRPC6 showing decreased expression in shRNA-TRPC6 treated mice. (*n* = 3, *P* < 0.05; *t*-test). Data were presented as mean ± SEM. *p < 0.05, **p < 0.01. Scale bar: 10 μm.

As in the hippocampus, the TRPC6 mainly localizes in the DG (Kim et al., 2013; Nagy et al., 2013), we infused the shRNA-TRPC6 into the DG mediating TRPC6 knockdown. Prominent GFP fluorescence showed that shRNA-TRPC6 was successfully transfected into the DG in the 4th week after injection. The expression of TRPC6 in the DG area was remarkably reduced after shRNA-TRPC6 virus transduction when compared to the control vector (shRNA-control) (Figure 2A). Since TRPC6 knockout mice showed increased TRPC3 expression as an adaptive response (Dietrich et al., 2005), the response of TRPC3 expression was also explored in this study. However, injection of shRNA-TRPC6 had no effect on TRPC3 mRNA expression (Figure 2B). Meanwhile, TRPC6 mRNA and protein levels in the hippocampus were significantly decreased by shRNA-TRPC6 intervention, validating TRPC6 protein suppression (Figures 2B–D).

### Knockdown of TRPC6 in DG Neurons Impaired Hippocampus-Dependent Memory in Mice

**FIGURE 3:**
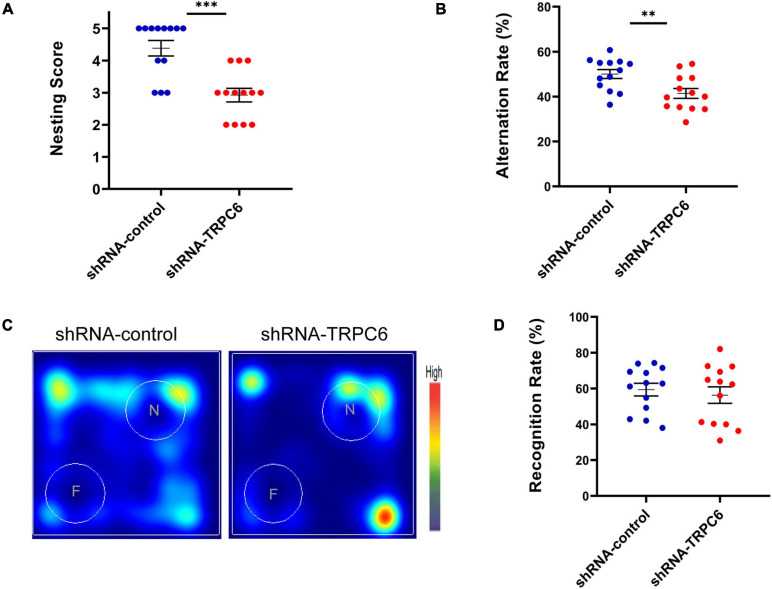
shRNA-TRPC6 injection affected hippocampal related-memory in mice. **(A)** shRNA-TRPC6 injected mice displayed impaired nest building abilities (***p < 0.001, *t*-test). **(B)** Scatter plot showing the percentage of correct spontaneous changes in both groups (**p < 0.01, *t*-test). **(C)** Representative heat maps showing the duration of time that the mice spent exploring the objects in NOR test. “F” and “N” represent the familiar object and the novel object, respectively. **(D)** There was no significant difference in recognition rate between the two groups. (*p* = 0.603; *t*-test). Sample sizes for each group: *n* = 13. Data were presented as mean ± SEM.

It has been indicated that nest building task is a reliable indicator of hippocampal-mediated cognitive ability (Chung and Cummings, 2000; Deacon et al., 2002). As shown in Figure 3A, nesting score in shRNA-TRPC6 injected mice was significantly declined in comparison to shRNA-control injected mice. As memory can be a measure of cognitive ability, the effects of TRPC6 deficits were further evaluated by Y maze and NOR, two types of memory that are dependent on the hippocampus to different degrees. In the Y-maze spontaneous alternation task, we found a clear defect with a lower rate of correct spontaneous alternation in the shRNA-TRPC6 injected mice compared with the control group (Figure 3B). However, in the hippocampus-dependent spatial object recognition test, mice in the two groups showed similar result in the object discrimination, indicative of no alteration in short-term spatial memory by reduced TRPC6 expression in the DG area (Figures 3C,D). Taken together, these results suggest that down-regulation of TRPC6 expression in the DG specifically impairs hippocampus-dependent spatial working memory.

### Reduced Expression of TRPC6 in DG Neurons Impaired Social Recognition in Mice

**FIGURE 4:**
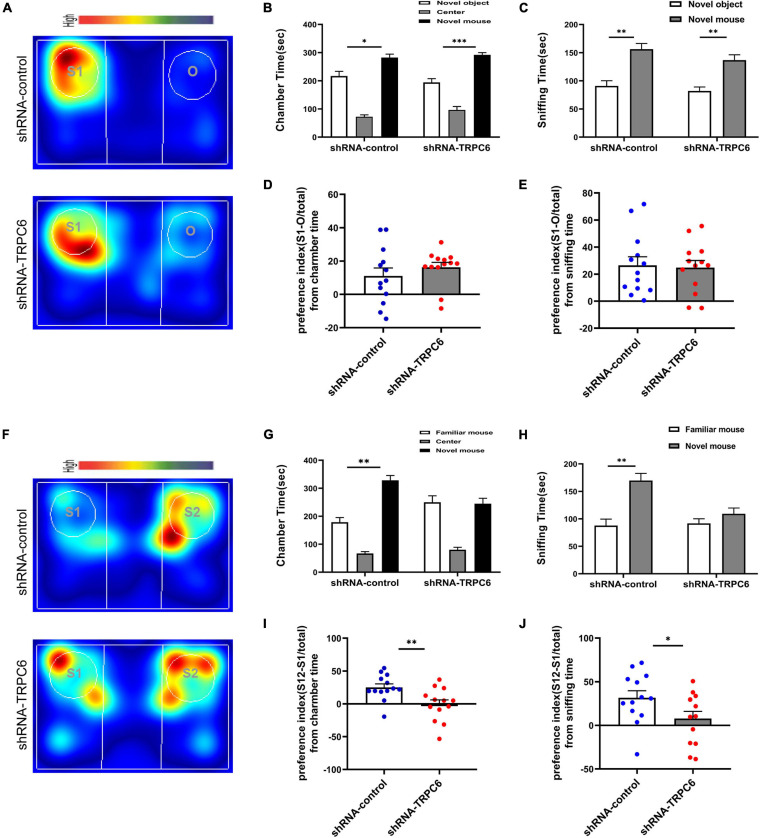
Reduced expression of TRPC6 caused impaired social memory but does not affect social function in mice. **(A,F)** Representative heat maps showing the total time and location of during the 10-min social approach task **(A)** and social novelty recognition **(F)**. Warmer colors (red) indicate longer time the mice spend exploring. “O” and “S1” represent object and novel mouse, “S1” and “S2” represent familiar mouse and novel mouse, respectively. **(B,C)** There was no difference between the two groups of mice in terms of chamber time and sniffing time. **(D)** Preference index (S-O/total) in chamber time. **(E)** Preference index (S-O/total) in sniffing time. **(G,H)** Control mice exhibited a significant preference for novel mouse compared with the familiar mouse, whereas mice injected with shRNA-TRPC6 exhibited a characteristic lack of social novelty as demonstrated by approximately equal amounts of time with the novel mouse and familiar mouse. **(I)** Preference index (S2-S1/total) in chamber time. **(J)** Preference index (S2-S1/total) in sniffing time. Sample sizes for each group: *n* = 13. Data were presented as mean ± SEM. *p < 0.05, **p < 0.01, ***p < 0.001.

Next, we examined the effects of down-regulation of TRPC6 expression in the DG on sociability and social recognition in mice using a three-chamber test. In the sociability test (Figure 4A), we found that subject mice in both groups spent more time in the side chamber containing a conspecific than in the side chamber containing an inanimate object (Figures 4B,C), and there was no significant difference between the two groups in terms of social preference index (Figures 4D,E), consistent with the definition of normal sociability in this task. As expected, mice injected with shRNA-control spent significantly (*p* < 0.01) more time exploring the novel conspecific in the social novelty recognition test, exhibited a clear preference for novel conspecifics. In contrast, mice injected with shRNA-TRPC6 abolished this preference and caused mice to spend similar amounts of time exploring mice that were novel or familiar (Figures 4F–H). The preference index derived from time in the chamber and sniffing shows significant difference between the two groups (Figures 4I,J). The above results suggest that down-regulation of TRPC6 expression impaired social recognition memory. These observations suggest that mice injected with shRNA-TRPC6 display intact sociability but lack social novelty preference.

### Reduced Expression of TRPC6 in the DG of Hippocampus Affected Exploratory Behavior of Mice

**FIGURE 5:**
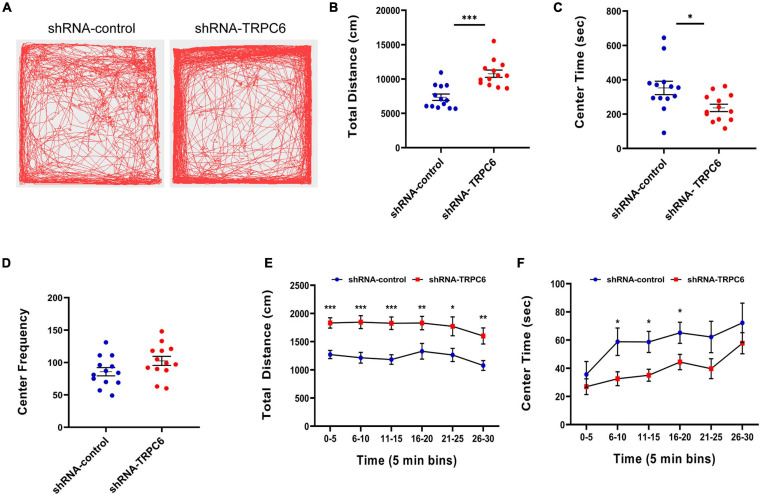
Down-regulation of TRPC6 affected exploratory abilities and anxiety-like behavior in mice. **(A)** Representative traces displaying the total distance traveled over the 30-min period by the experimental mice between the two groups in the open-field test. **(B)** Scatter plot showed a significant difference between the two groups in terms of total distance. **(C)** Mice in the shRNA-TRPC6 treated group spent less time in the centre than the control group. **(D)** Frequency of access to the central area. **(E)** The total distance over 30 min in 5-min bins. **(F)** The center time over 30 min in 5-min bins. Sample sizes for each group: *n* = 13. Data were presented as mean ± SEM. *p < 0.05, **p < 0.01, ***p < 0.001.

The open field test measures novelty-induced locomotion and anxiety-like behavior (Ruzicka et al., 2018). In this test, mice injected with shRNA-TRPC6 showed a significant increase in the distance traveled compared to mice in the shRNA-control group (Figures 5A,B,E), suggesting that mice injected with shRNA-TRPC6 display increased levels of novelty-induced locomotion. The amount of time spent in the center of the field over 30 min did significantly differ between mice in two group (Figure 5C), suggesting that mice injected with shRNA-TRPC6 have abnormal levels of anxiety-like behavior in the open field, but no difference in the frequency of the central area (Figure 5D). During the 6–20 min time period, we noticed there was a difference in the central area time of mice in the shRNA-control group compared to the shRNA-TRPC6 group (Figure 5F), suggesting reduced TRPC6 expression had a temporary effect on anxious behavior in these mice.

### Reduced Expression of TRPC6 in DG Neurons Impaired Long-Term Spatial Learning of Mice

**FIGURE 6:**
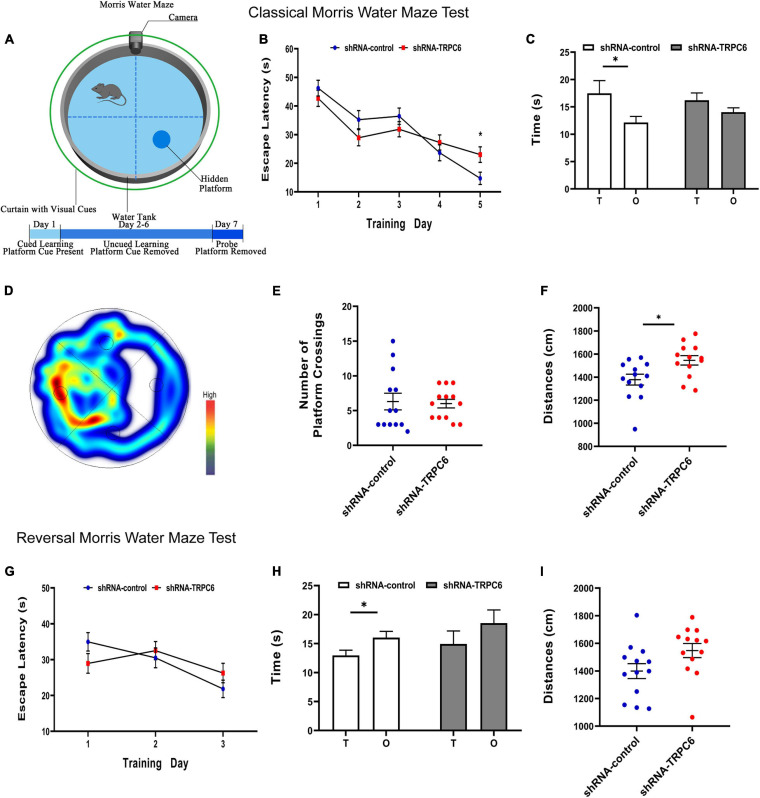
Down-regulation of TRPC6 affected the spatial learning in the MWM test. **(A)** Experimental design in the MWM test. **(B,G)** Plots showing the average escape latencies of mice in the hidden platform test during the training days. Repeated measures ANOVA were used. *p < 0.05. **(C,H)** Time spent in target quadrant (T) and opposite quadrant (O) in the probe test. *p < 0.05. **(D)** Heat map of shRNA-TRPC6 treated mice during the navigation trials. **(E)** The number of platform crossings shown in the scatter plot. **(F,I)** Scatterplots showing the distances by mice in the two groups during the probe test. Sample sizes for each group: *n* = 13. Data were presented as mean ± SEM.

The morris water maze was used to evaluate the effect of down-regulation of TRPC6 expression in the DG of hippocampus on long-term spatial learning and memory in mice (Figure 6A). On the first day of the experiment, to exclude the effects of mouse vision and water fear, we performed platform-visible exclusion training. In the position navigation experiment, the mean escape latency of mice searching for a hidden platform decreased with increasing days of training (two-way ANOVA: days: *p* < 0.001) (Figure 6B). In addition, during the learning phase, shRNA-TRPC6-injected mice had a significantly longer escape latency on day 5 (day5: *p* < 0.05) than that in the mice injected with shRNA-control. At the end of the acquisition phase, the cryptic platform was removed and a probe test was performed on day 7 to assess the mice’s memory recall. The probe test showed that shRNA-TRPC6 injected mice spent similar time in the target quadrant compared to the shRNA-control injected mice (Figures 6C,D). Moreover, there was a notable difference between the time spent in target quadrant and opposite quadrant of the shRNA-control injected mice, but no significant difference was observed in the shRNA-TRPC6injected mice (Figures 6C,D). We noticed that no differences were observed in the number of times they crossed the platform (*p* > 0.05) (Figure 6E). During the test, the shRNA-TRPC6 treated mice swam faster than the mice in the shRNA-control group, while the same difference in total swimming distance occurred between the two groups of mice (Figure 6F). These results suggest that down-regulation of TRPC6 expression affects the spatial learning and reference abilities of mice and also has an impact on aspects of cognitive plasticity in mice.

Furthermore, the cognitive flexibility of these mice was assessed by a subsequent reverse water maze test in which hidden platforms were moved to the opposite quadrant. Again, the mean escape latency of mice seeking hidden platforms decreased progressively with increasing days of training (two-way ANOVA: days: *p* < 0.001) (Figure 6G). In reversal training, no difference in the duration of the 3-day escape latency was observed between the two groups of mice. In the fourth day probe test, the results showed that mice in the shRNA-control group spent more time in the opposite quadrant than in the target quadrant, however mice in the shRNA-TRPC6 group showed an even presence across quadrants and no difference in total swimming distance (Figures 6H,I). These data suggest that mice in both groups were slower to erase memory of the original target and relearn the location of the new target, while mice in the shRNA-TRPC6 group exhibited memory confusion to some degree.

### RNA-Seq Results Showed That shRNA-TRPC6 Injected Mice Exhibited Genetic Alterations Associated With Synaptic Development

**FIGURE 7:**
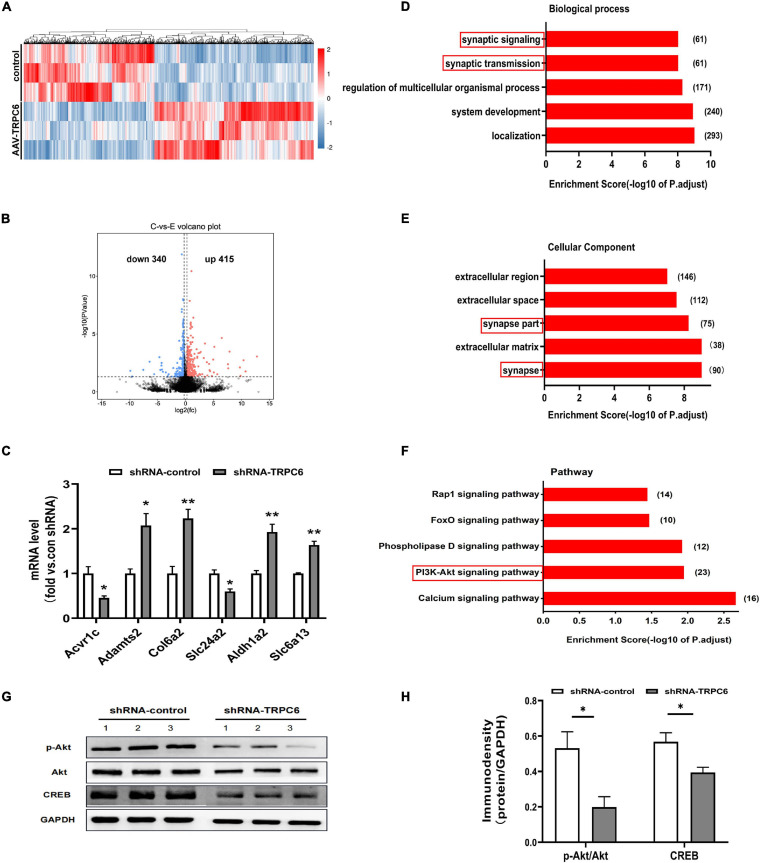
Down-regulation of TRPC6 altered gene expression associated with synaptic structure and function. **(A)** Heatmap of significantly up or downregulated genes in two groups. **(B)** A volcano plot showing differentially expressed genes in hippocampus of shRNA-control and shRNA-TRPC6 treated mice. Dashed line represents *p* < 0.05. Significantly up-regulated genes are shown in red for 415 genes, and significantly down-regulated genes are shown in blue for 340 genes, with down-regulated and up-regulated genes above | logFC| > 0.58 threshold. **(C)** Validation of gene expression in hippocampus of shRNA-control and shRNA-TRPC6 treated mice by qRT-PCR. **(D,E)** Enriched top five GO pathway in biological process and cellular component. The notable pathways which contain several pathways related to synaptic developmental function. The numbers listed next to the bars represent the number of differential genes in each pathway. The former significant pathway involved in synaptic developmental function is highlighted in red frame. **(F)** Five of the first twenty KEGG pathways were selected for significant enrichment differences, where the PI3K-Akt signaling pathway was found to be associated with cell survival and synaptic development has been highlighted in red frame. **(G)** Protein levels of Akt, pAkt, and CREB in the hippocampus isolated from shRNA-control or the shRNA-TRPC6 treated mice. **(H)** shRNA-TRPC6 treated mice displayed low levels of pAkt/Akt, and CREB in the hippocampus. Sample sizes for each group: *n* = 3. Data were presented as mean ± SEM. *p < 0.05, **p < 0.01.

To further elucidate the molecular mechanisms of cognitive dysfunction in shRNA-TRPC6 injected mouse DG, we used RNA-seq to identify DEGs in the hippocampus. A total of 755 genes were detected as differentially expressed (DE) when the false discovery rate (*p*-value) was less than 0.05 and the difference in expression levels was greater than 1.2. Among these genes, there were 415 up-regulated genes and 340 down-regulated genes (Figures 7A,B). We also selected some genes for validation and the results were consistent. Compared with the shRNA-control group, the mRNA expression levels of *Acvr1c and Slc24a2* were significantly decreased, while those of *Adamts2, Col6a2*, *Aldhla2*, and *Slc6a13* were significantly increased in the shTRPC6 injected group (Figure 7C). To identify the biological processes or cellular component enriched in the active modified transcripts, we performed gene ontology analysis using David bioinformatics resources^1^. In the biological process class, the top enriched clusters included those involved in localization, system development, regulation of multicellular organismal processes, synaptic transmission, synaptic signaling (Figure 7D). In the cellular component category, the top enriched clusters contain synapse, extracellular matrix, synapse part, extracellular space, and extracellular region (Figure 7E). It was previously found that TRPC6 is involved in the synapse development and (Tai et al., 2008), not surprisingly, GO analysis based on the DEGs list revealed a significant difference between the two groups in the genes related to synapse structure and function. Additionally, KEGG analysis showed that most of the DEGs were significantly enriched for the term calcium signaling pathway, PI3K-Akt signaling pathway, phospholipase D signaling pathway, FoxO signaling pathway, and Rap1 signaling pathway (Figure 7F). PI3K-Akt signaling pathway has been indicated to be associated with cell survival and synaptic development (Tai et al., 2008; Singh et al., 2017). As expected, the protein levels of pAkt and its target *CREB* were significantly decreased in the hippocampus of shTRPC6 injected group (Figures 7G,H).

### Repression of TRPC6 in the Hippocampal DG Region Altered Dendrite Spines of Granule Neurons

**FIGURE 8:**
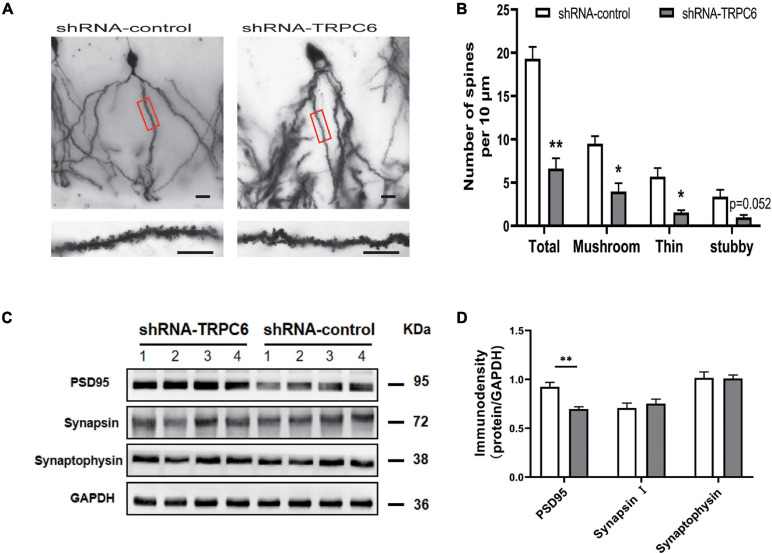
Dendritic spines of DG neurons and PSD95 in hippocampus in shRNA-TRPC6 treated mice were significantly decreased. **(A)** Photomicrographs of DG neurons dendrites taken at 20x power, dendritic branches in red panels are magnified at 100x power. **(B)** Quantification of dendritic spines of DG neurons per 10 μm branch showing decreased spine density in the shRNA-TRPC6 treated mice, quantification of each spine type showing immature development trend in shRNA-TRPC6 mice (*n* = 3, 3, *t*-test). **(C,D)** Down-regulation of TRPC6 decreased the levels of PSD95 in hippocampus extracts measured by western blotting and the quantitative analyses (*n* = 4 each group). Data were presented as mean ± SEM. *P < 0.05, **P < 0.01. Scale bars: 40 μm.

We performed Golgi staining to examine whether shRNA-TRPC6 treatment affected the dendrite spines of dentate granule cells (Figure 8A). It was found that decreased expression of TRPC6 resulted in a significant decrease in the dendrite spine density of DG granule neurons. Meanwhile, the dendrite spine densities of two types, mushroom and thin, were significantly decreased, while the dendrite spine densities of stubby type also showed a trend of decrease (Figure 8B). Thus, TRPC6 deficiency leads to structural changes of DG granule neurons. Next, we further performed western-blotting experiments to compare synaptic components at the protein level. Previous studies have shown that TRPC6 is localized at excitatory postsynaptic sites and co-localized with PSD95 (Zhou et al., 2008). As seen from our results, PSD95 protein expression was significantly reduced, while synapsin and synaptophysin expression were not significantly different between two groups (Figures 8C,D). These data further suggest that the reduction of TRPC6 alters neuronal synapses in the hippocampus region, which has an impact on cognitive function and learning memory in mice.

## Discussion

In this study, our results provided evidence that TRPC6, in the granule cells of DG is involved in the hippocampus related cognitive function. Injection of shRNA-TRPC6 into the hippocampal DG region knocked down TRPC6 mRNA and protein levels in adult male mice. Knocking down TRPC6 in the DG neurons impaired learning and memory performance, as assessed by the escape latency in the MWM, the correct spontaneous alternation in the Y-maze test, social recognition memory in the three-chamber test. Furthermore, viral intervention of TRPC6 expression in DG resulted in a reduced density and morphological abnormalities of dendritic spines of DG granule neurons, which may affect synaptic function. These results suggested that knocking down TRPC6 in the DG may have a disadvantageous effect on learning and memory in mice.

TRPC6 is abundantly expressed mainly in the DG of hippocampus (Chung et al., 2006; Tai et al., 2008; Kim et al., 2013). In most mammalian species, the DG has been proposed to serve several memory-aiding functions, including pattern separation, pattern completion, novelty detection, binding of information to spatial context and working memory (Leutgeb et al., 2007; Anacker et al., 2018; Senzai, 2019; Hainmueller and Bartos, 2020). Here, a long-acting AAV used in the hippocampal DG region to down-regulate TRPC6 expression was injected into mice. We confirmed the efficacy of this virus based on low TRPC6 expression in the DG 28 days after AAV injection.

The behavioral changes in the shRNA-TRPC6 injected mice were highly selective indicating specific functional roles for TRPC6 expressed in DG neurons. Mice injected with shRNA-TRPC6 displayed greater impairment on the nesting assay compared to control animals. Down-regulation of TRPC6 reduced the percentage of correct spontaneous alternations in the Y-maze test, indicating short spatial memory was hindered. Enhanced performance in spatial learning and memory has been found in the TRPC6 transgenic mice. Down-regulate of TRPC6 did not lead to changes in the short-term memory in NOR test and spatial memory in the MWM, although the shRNA-TRPC6 treated mice had a significantly longer escape latency time on the last day indicating mild deficit in the spatial learning. Interestingly, in the open field test, the shRNA-TRPC6 treated mice exhibited an enhancement of locomotor activity compared to that in the control group. Moreover, animals in the shRNA-TRPC6 treated group spent less time in the central area of the open field than that in the control group, implying that shRNA-TRPC6 in the DG generated a range of specific behavioral pattern including increased locomotor activity and anxiety-like behavior. We noticed that down-regulation of TRPC6 expression significantly affected the social memory of the mice without altering sociability, indicating a specific deficit in social recognition.

Interestingly, the behavioral changes in the shRNA-TRPC6 injected mice remained significantly different from shRNA-TRPC6 injected mice at 8 months of age. The shRNA-TRPC6 injected mice still showed deficits in the nesting task and working memory assessed by Y-maze, although the two groups did not differ in the NOR experiment (Supplementary Figures 1B–D). Meanwhile, we found that mice after down-regulation of TRPC6 expression exhibited social impairment and social memory cognitive impairment at 8 months old (Supplementary Figures 1E–L). Furthermore, we found a significant decrease in TRPC6 expression in the hippocampus of shRNA-TRPC6 injected mice at 8 months of age, compared to control mice of the same age (Supplementary Figure 1A). Overall, down-regulation of TRPC6 expression in the hippocampal DG region of adult-aged mice by way of AAV virus injection leads to similar or worse cognitive impairment.

Previous studies have found that TRPC6 is involved in synapse formation (Li et al., 2005; Tai et al., 2008; Zhou et al., 2008; Leuner et al., 2013), not surprisingly, GO analysis based on the DEGs revealed shRNA-TRPC6 treated mice had a significant enrichment in biological process related to synaptic signaling and transmission, and in cellular component related to synaptic part. This indicates that knocking down TRPC6 in the DG led to a change in transcriptional profile highly associated with the synaptic structure and function. The structural integrity of synapses is crucial for the transmission of information between neurons (El-Husseini et al., 2000; Biederer et al., 2017; Borges-Merjane et al., 2020). PI3K phosphorylates Akt, and the activated Akt can further phosphorylate CREB, which plays important roles in synapse formation, and learning and memory (Tai et al., 2008; Marsden, 2013; Singh et al., 2017). KEGG analysis showed PI3K-Akt signaling pathway were significantly down-regulated in the hippocampus of shRNA-TRPC6 treated mice. The decreased protein levels of pAkt and CREB were verified in the hippocampus of shRNA-TRPC6 injected group might interpret the underlying mechanism of TRPC6 involved in the synaptic structure and function. Additionally, dendritic spines mainly form the postsynaptic sites of excitatory neurotransmission and are critical for maintaining synaptic function and plasticity (Russo et al., 2010; Sala and Segal, 2014). Alterations in the morphology of dendritic spines can affect synaptic signaling transmission, synaptic plasticity and individual learning and memory abilities (Hoover et al., 2010; Chang et al., 2014). Golgi staining showed a significant reduction in dendritic spine density in the shRNA-TRPC6 group, as well as a decrease in post-synaptic protein levels, compared to the control group.

The process of separating the components of a complex memory and converting them into a unique and non-confusing representation of the memory becomes pattern separation (Buzsáki and Llinás, 2017). The DG of the hippocampus performs pattern separation by activating different groups of neurons as the animal’s environment changes, with each combination of neurons responsible for memory of one environment (Ramirez et al., 2013). When neurons show abnormalities such as reduced synaptic and dendritic spines, this will affect the memory function of the DG (Cheng et al., 2021). In this study, mice in the shRNA-TRPC6 group were unable to discriminate between novel and familiar mice in three-chamber test and interestingly, the two groups did not differ in the new object recognition experiment, while the mice had impaired spatial learning and working memory. This result may be due to the fact that granule cells of DG manifest cognitive impairment in mice due to decreased levels of synaptic and dendritic spines leading to pattern separation dysfunction.

The down-regulation of TRPC6 protein in the DG region on cognitive impairment has been demonstrated in this study. Existing studies have shown that cognitive impairment is the most common and challenging syndrome in clinical practice in Alzheimer’s disease (Lu et al., 2017; Rabin et al., 2017), and that blood cell TRPC6 mRNA levels are reduced in patients with Alzheimer’s disease and mild cognitive impairment (Lu et al., 2018; Chen et al., 2019). Specifically, our laboratory previously detected abnormal expression of TRPC6 in the cerebellum of dystonia-like behaviors and motor dysfunction of autistic BTBR mice (Xiao et al., 2020). The phenotype of cognitive dysfunction identified in this study is similar to that of Alzheimer’s disease patients, the abnormalities in motor ability of the mice were also similar to those of autistic BTBR mice and this finding may provide new therapeutic ideas for the improvement of patients with cognitive impairment. To date, the important role of TRPC6 protein has been identified in studies of many neurological disorders such as stroke, ASD and epilepsy (Griesi-Oliveira et al., 2015; Zeng et al., 2015; Liu et al., 2020), suggesting the importance of TRPC6 in maintaining normal neuronal function. In fact, TRPC6 channels have been studied as an emerging area of research as a molecular target for neuroprotective agents in Alzheimer’s disease and cerebral ischemia. Over-expression of TRPC6 has been shown to increase dendritic spine density, rescue mushroom-type dendritic spine loss in mouse models of AD, and protect against neuronal loss. The main TRPC6 agonists currently in use for the treatment of endogenous TRPC6 expression reduction in mouse models are endogenous diacylglycerols (DAGs) (Montecinos-Oliva et al., 2014), hyperforin (Leuner et al., 2007, 2013), resveratrol (Lin et al., 2013) and others, have shown positive effects. It is feasible and attractive to explore potential therapeutic strategies targeting TRPC6 for cognitive-related functions in the DG region.

## Conclusion

We demonstrated that spatial learning, working memory and social recognition memory were impaired in mice when TRPC6 in the DG is declined. We also observed that knocking down TRPC6 lead to deficits in dendrite spines. All of above confirm that there is an association between reduced expression of TRPC6 and impaired cognition ability.

## Data Availability Statement

The datasets presented in this study can be found in online repositories. The names of the repository/repositories and accession number(s) can be found in the article/Supplementary Material.

## Ethics Statement

The animal study was reviewed and approved by Animal Experiment Committee of Laboratory Animal Center of Army Medical University. Written informed consent was obtained from the owners for the participation of their animals in this study.

## Author Contributions

RXX designed and implemented the experiments, collated and analyzed the data, and wrote the original manuscript. RX, ZW, and KL contributed to conception and design of the study. YL, TL, and YC monitored the status of the mice and collected the samples. YL and CW contributed to the collection of data for quantification and statistical analysis. XF reviewed the experimental plan, provided technical and financial support, and revised the manuscript. All authors contributed to the article and approved the submitted version.

## Conflict of Interest

The authors declare that the research was conducted in the absence of any commercial or financial relationships that could be construed as a potential conflict of interest.

## Footnotes

1 https://david.ncifcrf.gov/

## Abbreviations

AAV: adeno-associated virus
ASD: autism spectrum disorders
BSA: bovine serum albumin
CNS: central nervous system
CREB: the cAMP (adenosine 3 ′,5 ′ -cyclic monophosphate) response element-binding protein
DAGs: diacylglycerols
DEGs: differential expression genes
DG: dentate gyrus
ECL: enhanced chemiluminescence
FDR: false discovery rate
GAPDH: glyceraldehyde-3-phosphate dehydrogenase
GO: Gene Ontology
NOR: novel object recognition
MWM: morris water maze test
OFT: open-field test
PBS: phosphate buffered saline
PFA: paraformaldehyde
PSD95: postsynaptic density-95
PVDF: polyvinylidene fluoride
RIPA: radio immunoprecipitation assay
SEM: standard error of the mean
shRNA: short hairpin RNA
TRPC: transient receptor potential canonical channel.
