# Supplementary material for: AAV Delivery of shRNA Against TRPC6 in Mouse Hippocampus Impairs Cognitive Function
Source: Front Cell Dev Biol. 2021 Jul 13;9:688655. doi: 10.3389/fcell.2021.688655 (PMC8313999; doi:10.3389/fcell.2021.688655)
Supplement: Supplementary file 1 [file Table_1.DOCX]

Supplementary Material

# Supplementary Figure and Figure legend

## Supplementary Figure


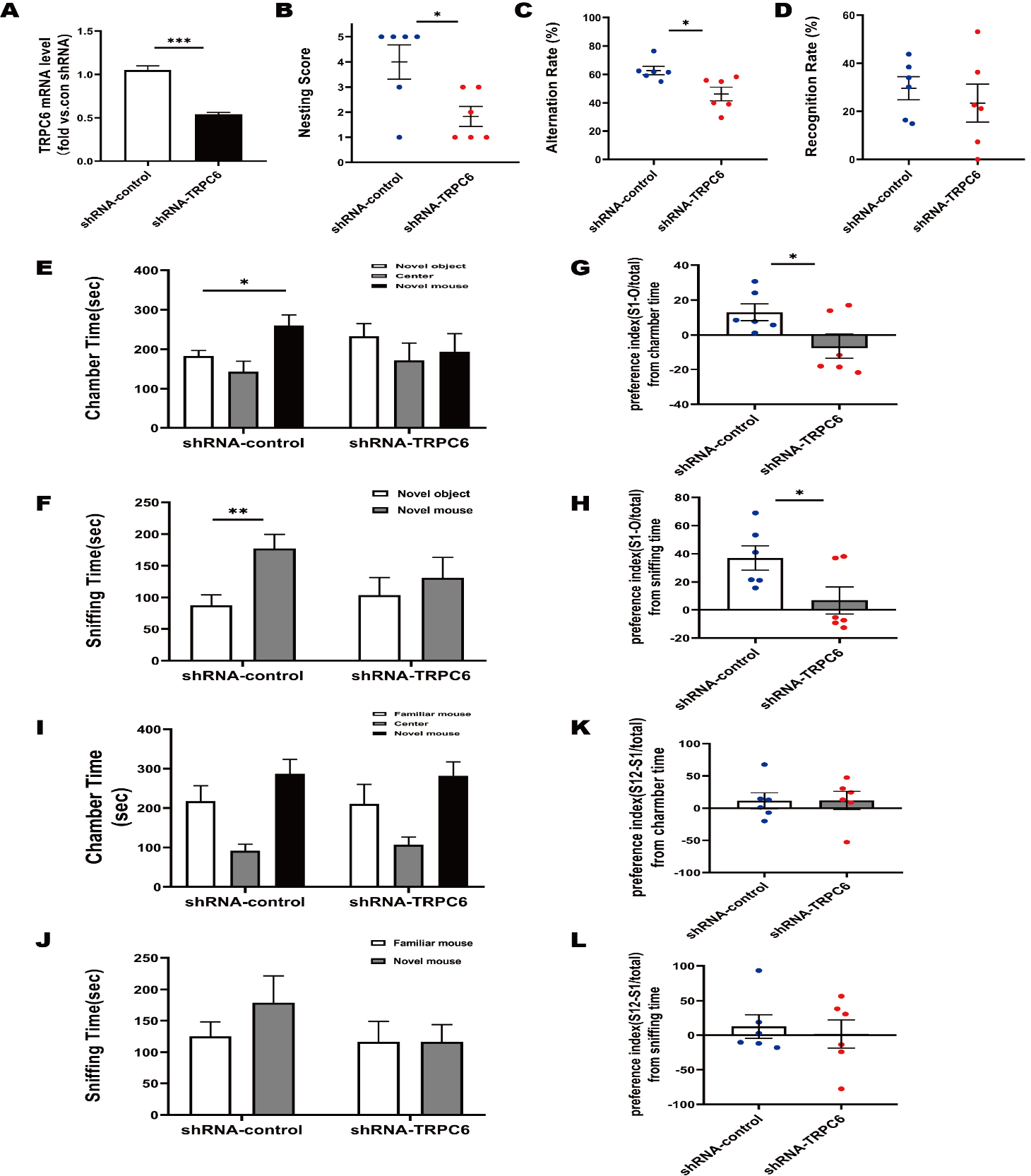


**Supplementary Figure 1.** **shRNA-TRPC6 injected mice at 8 months of age show deficits in cognitive performance.**

Two groups of mice injected with the shRNA-TRPC6 or shRNA-control virus were subjected to behavioral assays at 8 months of age. (A) TRPC6 mRNA expression in 8-month-old shRNA-control and 8-month-old shRNA-TRPC6 treated mice by qRT-PCR. Sample sizes for each group: n = 3. *** *p* < 0.001. (B)Nest building task. (C) Y-maze test. Scatter plot showing the percentage of correct spontaneous changes in both groups. (D) Novel object recognition (NOR). There was no significant difference in recognition rate between the two groups. (E-L) Three-chamber sociability test. There were significant differences between the two groups of mice in terms of chamber time and sniffing time in E and F. (G) Preference index (S−O/total) in chamber time. (H) Preference index (S−O/total) in sniffing time. (I and J) No differences were observed between the two groups of mice between novel mouse and familiar mouse. (K) Preference index (S2-S1/total) in chamber time. (L) Preference index (S2-S1/total) in sniffing time. Sample sizes for each group: n = 6. Data were presented as mean ± s.e.m. * *p* < 0.05, ** *p* < 0.01.
